# One-year efficacy of the LIFESTYLE intervention in improving diet, physical activity and reducing alcohol use in overweight people with severe mental illness: multicentre randomised controlled trial

**DOI:** 10.1192/bji.2025.10076

**Published:** 2026-11

**Authors:** Mario Luciano, Pierluigi Catapano, Gaia Sampogna, Bianca Della Rocca, Matteo Di Vincenzo, Federico Maj, Enrico D’Ambrosio, Antonio Rampino, Mario Amore, Pietro Calcagno, Francesca Pacitti, Rodolfo Rossi, Barbara Carpita, Liliana Dell’Osso, Giorgio Di Lorenzo, Alberto Siracusano, LIFESTYLE Working Group, Andrea Fiorillo

**Affiliations:** 1 Department of Psychiatry, University of Campania Luigi Vanvitelli, Naples, Italy; 2 Department of Basic Medical Science, Neuroscience and Sense Organs, University of Bari Aldo Moro, Bari, Italy; 3 Section of Psychiatry, Department of Neuroscience, Ophthalmology, Genetics and Infant-Maternal Science, University of Genoa, Genoa, Italy; 4 Department of Biotechnological and Applied Clinical Sciences, University of L’Aquila, L’Aquila, Italy; 5 Department of Clinical and Experimental Medicine, University of Pisa, Pisa, Italy; 6 Department of System Medicine, University of Rome Tor Vergata, Rome, Italy

**Keywords:** Psychoeducation, lifestyle, severe mental disorders, randomised controlled trial, rehabilitation and social psychiatry

## Abstract

**Background:**

Unhealthy eating patterns, physical inactivity and alcohol misuse are commonly reported by individuals with severe mental illness (SMI) and significantly contribute to premature mortality. People with SMI could benefit from psychoeducational interventions focused on lifestyle modification.

**Aims:**

To evaluate the effectiveness of the LIFESTYLE programme to improve dietary habits and physical activity levels and reduce alcohol use in individuals with SMI versus controls receiving a less structured psychoeducational programme (Italian Ministry of University and Research, trial registration number: 2015C7374S).

**Method:**

This multicentre randomised controlled trial (RCT) was conducted across six Italian universities and included 401 participants diagnosed with SMI, randomly allocated to either the test group or a comparison group.

**Results:**

At 1-year follow-up, generalised estimating equations showed that the trial intervention boosted the likelihood of higher weekly metabolic equivalents of task (METs) expended on total activity (odds ratio 1.43, 95% CI 1.08–1.89; *p* < 0.01), on walking (odds ratio 1.50, 95% CI 1.18–1.90; *p* < 0.001) and on moderate activity (odds ratio 1.85, 95% CI 1.24–2.77; *p* < 0.01). Improvements in dietary habits included increased intake of fish (odds ratio 1.67, 95% CI 1.45–1.97; *p* < 0.05), fresh fruit (odds ratio 1.36, 95% CI 1.05–1.76; *p* < 0.05) and vegetables (odds ratio 1.91, 95% CI 1.56–1.96; *p* < 0.05), along with reduced junk food consumption (OR = 0.81, 95% CI 0.63–0.99; *p* < 0.05) and daily alcohol use (odds ratio 0.70, 95% CI 0.52–0.95; *p* < 0.05).

**Conclusions:**

The LIFESTYLE intervention proved effective in promoting healthier lifestyles among individuals with SMI, with sustained benefits at 1 year. This structured programme could be a valuable addition to routine mental healthcare.

Lifestyle behaviours are receiving increasing interest among clinicians and researchers in mental health owing to their proven potential to reduce the incidence of chronic and disabling physical illnesses and mortality in individuals with severe mental illness (SMI).^1^ Healthy habits represent a pattern of behavioural choices and include adequate daily physical activity, balanced diet, discontinuation of alcohol consumption and smoking cessation. Healthier living is usually associated with a reduced incidence of physical illnesses, including cancer and cardiovascular, gastrointestinal and bronchopulmonary diseases, ultimately resulting in reduced mortality, increased life expectancy and decreased disability.^1^ Physical activity and a healthy diet are fundamental components of a healthy lifestyle, since they can positively influence both physical and mental health, lowering anxiety and depressive symptoms, improving cognition and enhancing psychological well-being.^2^ In particular, physical activity is a protective factor even when other risk factors for chronic diseases are present.^3^ Individuals with SMI, including psychotic disorders, major depression and bipolar disorder, are more likely to adopt unhealthy behaviours compared with the general population.^4^ For instance, they are less likely to undertake 2.5 h of medium to intense exercise every week.^5^ Additionally, these people often report unhealthy dietary patterns, including low consumption of vegetables and high intake of processed food, leading to poor nutritional status, with negative consequences on physical and mental health.^6,7^

Furthermore, alcohol misuse is frequently reported among people with SMI, even when a diagnosis of alcohol-related disorder is not present, with a detrimental impact on physical and mental health, increasing the risk of exacerbating the symptoms of a pre-existing mental or physical illness.^8^

Finally, adopting unhealthy behaviours in daily life significantly increases the likelihood of physical comorbidities, accounting for a significantly earlier mortality than in the non-clinical population.^9^ In particular, individuals with SMI are known to be more prone to develop metabolic syndrome. Although this is usually blamed on antipsychotic medications, other disease-specific factors can be considered, including cognitive impairment, reduced psychosocial functioning, social isolation and self-stigma, which can reduce patients’ autonomy to make decisions about their physical health.^10^

Given the importance of healthy daily habits, specific psychoeducational interventions aimed at improving these behaviours have been tested.^11^ Several randomised controlled trials (RCTs) have shown that these interventions can have a positive impact on the course of psychiatric disorders and cardiovascular, metabolic and other physical illnesses.^12^ In particular, interventions targeting specific goals, such as smoking cessation or physical activity, or increasing patients’ awareness of the basic aspects of healthy lifestyles, can decrease the probability of developing chronic diseases and improve general health.^13^ Although several trials have suggested that lifestyle interventions can be effective in improving health behaviours in people with SMI, most of these studies have important methodological limitations. A recent systematic review found that 64% of studies were at high risk of bias,^2^ with limitations such as insufficient sample sizes, short follow-up periods, single-centre designs, flaws in the randomisation process, absence of masking (‘blinding’), lost data, inadequate reporting of outcomes, statistical divergency and absence of active control groups.^14^ These limitations reduce the strength of the available evidence and justify the need for further rigorous evaluation through large multicentre RCTs. In addition, even when efficacy is demonstrated, the implementation of interventions in routine practice remains limited and deserves separate consideration.

## Aims

Given these shortcomings, we performed a multicentre RCT to assess the efficacy of a lifestyle programme specifically developed for people with SMI, combining classic psychoeducation, a cognitive–behavioural approach and health coaching. Unlike most studies in this field, we compared the experimental intervention (LIFESTYLE) with a control group receiving a generic psychoeducational intervention.

In this paper we analyse the effectiveness of the 5-month LIFESTYLE programme in improving dietary habits and physical activity and reducing alcohol use at 1-year follow-up.

## Method

We analysed data from the LIFESTYLE study (Italian Ministry of University and Research, trial registration number: 2015C7374S), a randomised controlled trial conducted in the out-patient care departments of the universities of Pisa, Bari, Rome Tor Vergata, Genoa, L’Aquila and Naples (Naples also acted as the coordinating centre). Each centre had to recruit 70 participants, randomly assigned to the experimental or control condition by the coordinating centre at a 1:1 ratio, stratified by sociodemographic characteristics. Before the baseline assessment, the LIFESTYLE staff contacted a statistician at the coordinating centre to obtain the randomisation code. Clinician raters at each centre and statisticians at the coordinating centre were masked to group allocation, whereas participants and the clinicians delivering the interventions were aware of assignment. Full methodological details are available in the pre-published protocol.^15^

Participants in both groups received pharmacological treatment throughout the study, according to most recent National Institute for Health and Care Excellence (NICE) guidelines for the relevant disorder. No other psychosocial interventions were offered to participants during the study, and concomitant medications were recorded and included in multivariable analyses. As a result, pharmacological treatment was neither manipulated nor standardised as part of the study protocol.

The following criteria were selected for inclusion: (a) age, ranging from 18 to 65 years; (b) main diagnosis of an SMI (schizophrenia or other psychotic disorder, major depression or bipolar disorder) in accordance with DSM-5 criteria and validated by the Structured Clinical Interview for DSM-5 (SCID-5); (c) capacity to offer informed consent; (d) having a body mass index (BMI) ≥25.

Participants were excluded if they matched any of the following criteria: (a) inability to engage in moderate physical activity (e.g. unstable cardiovascular disease or severe cardiopulmonary conditions that contraindicate exercise – individuals with stable cardiovascular or metabolic comorbidities were not excluded); (b) pregnancy or breastfeeding; (c) hospital admission in the 3 months prior to enrolment.

All procedures in this research adhere to the ethical guidelines of the appropriate national and institutional human experimentation committees and the 1975 Helsinki Declaration, updated in 2013. All procedures engaging humans received approval from the Ethics Committee of the coordinating centre (University of Campania Luigi Vanvitelli, Naples) in January 2017 (approval number: protocol number 64). The study also gained approval from the ethical boards at each participating location. All participants gave their written informed consent.

At enrolment, participants were given a full explanation of the objectives, goals, format, structure and scheduling of both interventions. Participants received information leaflets at the end of each session. Treatment fidelity across the various sites was ensured through manuals for both treatments, as well as ongoing phone conversations and on-site supervision.

This study follows the latest CONSORT guidelines for reporting randomised trials with comparable groups.^16^ The completed checklist and flowchart can be found in the Supplementary Material, available online at https://doi.org/10.1192/bji.2025.10076.

### Experimental intervention

The LIFESTYLE intervention includes eight modules delivered every 7–10 days over 5 months, covering diet, physical activity and other lifestyle topics, following guidelines from the World Health Organization^17^ and European health associations.^18^ Psychoeducation, motivational interviewing and cognitive–behavioural therapy are the main ingredients of the newly developed intervention, which is delivered to groups of 5–10 patients.^19^ Each session encourages interaction and concludes with 20 min of moderate physical activity. In addition, mental health professionals support participants in setting and achieving healthy individualised lifestyle goals. Sessions were held in out-patient psychiatric facilities by trained mental health professionals. All facilitators underwent standardised training on the intervention prior to the trial and received ongoing supervision from the coordinating centre, to ensure fidelity across centres. Moreover, regular on-site visits and *in vivo* supervision were provided by facilitators at the coordinating centre. The full methodology has been described in greater detailed elsewhere.^15^

### Control intervention

The control intervention was a brief educational programme consisting of five modules delivered to groups of 5–10 patients in five weekly sessions over a period of 2 months. It focused on diet, early relapse signs, medication information, stress management and problem-solving. Manuals were used to guarantee consistent implementation across centres, and leaflets and written materials were distributed when relevant. Interaction among participants was facilitated using role-play and collaborative group exercises.

### Assessment time points and instruments

The study employed a single-blind design, with researchers and statisticians conducting the assessments unaware of group allocation. Participants were evaluated at the start of the study (T0) and after 6 months, 1 year and 2 years. For this paper we used data from the 1-year follow-up (T2). Findings from the 6-month analyses (T1) are available elsewhere.^12,20^

Primary diagnoses were confirmed using the Structured Clinical Interview for DSM-5 (SCID-5). Mental health was assessed using the validated versions of the following instruments: (a) the Brief Psychiatric Rating Scale (BPRS), which is a semi-structured interview with 24 items designed to assess psychopathology; (b) the Personal and Social Performance Scale (PSP), a single-item scale rated out of 100 points; (c) Cumulative Illness Rating Scale (CIRS).

To assess lifestyle habits, the following tools were used: (a) the abbreviated version of the International Physical Activity Questionnaire (IPAQ), a self-reported scale. Weekly metabolic equivalents of task (METs, minutes/week) expended were determined by multiplying the time spent (in minutes) on various activity categories (vigorous activity, moderate activity, walking) by their MET values (8.0, 4.0 and 3.3 respectively). Moderate activities include brisk walking, gardening and recreational cycling, whereas vigorous activities include running, aerobics and competitive sports. The total METs were the sum of scores from the walking, moderate and vigorous activities per week; (b) a 24-item Lifestyle Behaviors Questionnaire from the Italian National Institute of Health, a self-report survey that evaluates dietary patterns, smoking habits and alcohol use.

The inter-rater reliability of the PSP and BPRS was satisfactory (PSP: Cohen’s *κ* = 0.918; BPRS: *κ* = 0.835–0.972). There was complete consensus on the SCID-5 diagnosis (100%).

### Statistical analyses

All analyses were conducted under an intention-to-treat framework, using last observation carried forward imputation for missing data. Descriptive tables report completers, and generalised estimating equations (GEE) models include all randomised participants.

The normality of outcome variables was checked with Kolmogorov–Smirnov tests. All these variables were normally distributed, except for the IPAQ subscales. Descriptive statistics at the start and conclusion of the intervention included means and standard deviations, as well as interquartile ranges (IQRs) and medians for IPAQ subscales. The pre-published protocol specifies primary and secondary outcomes. BPRS subscales were analysed as secondary/exploratory outcomes,^15^ in accordance with the study protocol.

Variations in demographic and clinical traits between the groups at the start and after the intervention were evaluated using chi-squared tests or independent-sample *t*-tests. The effects of the interventions on the results after 1 year were evaluated using the Wilcoxon test and chi-squared test, as suitable.

Continuous variables were summarised using medians and IQRs rather than means and standard deviations, as several distributions (e.g. METs) were markedly skewed and included extreme values. This approach was prespecified in the statistical analysis plan to reduce the undue influence of outliers.

Variables that were found to be statistically significant in univariate analyses were entered into a GEE model. Time × group interaction terms probed the changes over time; their joint effects were tested by Wald tests. GEEs were corrected for age, study centre, medications, cognitive functioning, gender, diagnosis and pharmacological treatments. The dummy variables in the regression model covered mood stabiliser, antidepressant and antipsychotic medications, and depressive, bipolar and psychotic disorders. Covariates were adjusted with robust standard error estimates.

## Results

In total, 401 individuals consented to participate in the research and were assigned in a random manner to either the experimental group (206 participants) or the control group (195 participants).

The intervention was completed by 170 participants: 87 (out of 206) from the experimental group and 83 (out of 195) from the control group. At baseline, no significant statistical differences in sociodemographic characteristics, diagnoses or clinical severity assessed using the BPRS were found between those who dropped out of the study and those who completed it. Reasons for drop-out included: changes in the local mental health centre (30%); difficulties in attending sessions (27%); severe worsening of mental health (20%); low motivation (18%); declining to complete the assessments at 1-year follow-up (5%).

### Sociodemographic and clinical characteristics

The majority of participants were female (57%); participants had a mean age of 45.6 years (s.d. = 11.8) and an average of 11.7 years of education (s.d. = 2.9). Among the 401 enrolled participants, 43.3% were diagnosed with bipolar disorder, 29.9% with schizophrenia/schizoaffective disorder and 26.9% with major depressive disorder. In both groups, less than 50% of participants were living with a partner. Every participant was taking at least one psychotropic drug: 35% were on one, 39% on two, 21% on three and 5% on four different medications. No statistically significant differences were found between the two groups with respect to sociodemographic and clinical characteristics (Supplementary Table S1).

### Efficacy of experimental intervention

After 1 year, statistically significant changes were found in physical activity levels between the experimental and control groups (Table 1). In the experimental group, an increase was observed between T0 to T2 in vigorous activity METs (*p* < 0.01) and in moderate activity METs (*p* < 0.01). No statistically significant T0–T2 differences were found in the controls. In the experimental group, we also found an increase in walking activity METs (*p* < 0.001) and in total activity METs (*p* < 0.001). It is noteworthy that a reduction in walking (*p* < 0.01) and in total METs (*p* < 0.001) was observed in the control group. Changes in physical activity over 12 months are shown in Supplementary Fig. 2, which illustrates the increase in physical activity METs in the experimental group compared with controls.

**Table 1:** Changes in physical activity levels between initial assessment and 1-year follow-up across both groups Table 1 long description.

Activity category	Experimental group		Control group	
	Baseline (T0) METS, minutes/week (*n* = 206)	1-year follow-up (T2) METS, minutes/week (*n* = 87)	Baseline METS, minutes/week (*n* = 195)	1-year follow-up (T2) METS, minutes/week (*n* = 83)
Vigorous activity				
Mean (s.d.)	280.2 (1195.9)	299.3 (791)	367.0 (1228.0)	111.3 (87.1)
Median (IQR)	0 (0)	300.0 (71)**	0 (0)	80 (145.0)
Moderate activity				
Mean (s.d.)	238.9 (800.5)	343.2 (995)	248.8 (1184.1)	77.9 (66.5)
Median (IQR)	0 (0)	345 (100.0)**	0 (0)	69.0 (100.8)
Walking				
Mean (s.d.)	922.4 (1475.7)	1194.8 (483.1)	719.4 (1325.1)	581.4 (385.1)
Median (IQR)	362 (1138.5)	1095.0 (559.3)***	264.0 (631.1)	225.0 (294.5)**
Total physical activity				
Mean (s.d.)	1441.5 (2449.7)	1837.4 (1700.0)	1335.2 (2195.7)	770.6 (500.5)
Median (IQR)	519.5 (1745.2)	1736.5 (1350,4)***	541.0 (1745.3)	459.9 (344.3)***

METs, metabolic equivalents; IQR, interquartile range.

*p < 0.5; **p < 0.01; ***p < 0.001.

In summary, the intervention led to a clear increase in physical activity, particularly in vigorous and moderate activity METs, which are clinically relevant behaviours associated with better health outcomes.

Participants receiving the experimental intervention also improved their dietary habits (Table 2). The percentage consuming red meat never or once a week increased from 42.4% at baseline to 51.2% at 1-year follow-up (*p* < 0.05). Similarly, participants eating cured meat never or once a week increased from 47.1 to 65.1% (*p* < 0.01), and those consuming junk food never or once a week increased from 75.5 to 86.9% (*p* < 0.05). Additionally, participants eating fish over three times weekly/every day rose, from 38.4 to 47.1% (*p* < 0.05). In the experimental group we also found an increase in weekly intake of fresh fruit (over three times weekly/every day: from 76.7 to 88.4%; *p* < 0.01) and of vegetables (over three times weekly/every day: from 80.1% to 91.9%; *p* < 0.01). Last, weekly consumption of junk food decreased from 23.5 to 13.1% (*p* < 0.05). No statistically significant changes in dietary habits were observed in the control group.

**Table 2:** Changes in eating patterns between initial assessment and 1-year follow-up across both groups Table 2 long description.

	Experimental treatment		Control group	
	Baseline (T0) (*n* = 206), *n* (%)	1-year follow-up (T2) (*n* = 87), *n* (%)	Baseline (T0) (*n* = 195), *n* (%)	1-year follow-up (T2) (*n* = 86), *n* (%)
Bread				
Not at all/once weekly	14.6 (30)	10.6 (10)	16.7 (33)	15.7 (13)
Over three times weekly/every day	84.0 (176)	89.34 (76)	83.3 (162)	84.3 (73)
Pasta and rice				
Not at all/once weekly	10.3 (21)	7.1 (6)	9.4 (18)	7.1 (7)
Over three times weekly/every day	89.7 (182)	92.9 (80)	90.6 (174)	92.9 (79)
Red meat				
Not at all/once weekly	42.4 (86)	51.2 (44)^*^	48.4 (93)	47.7 (41)
Over three times weekly/every day	57.6 (117)	48.8 (42)	51.6 (99)	52.9 (45)
White meat				
Not at all/once weekly	33.0 (69)	31.8 (28)	33.0 (67)	35.7 (31)
Over three times weekly/every day	67.0 (137)	68.2 (58)	67.0 (136)	64.3 (55)
Legumes				
Not at all/once weekly	57.1 (116)	52.3 (45)	53.6 (103)	55.3 (47)
Over three times weekly/every day	42.9 (87)	47.7 (42)	46.4 (89)	44.7 (39)
Fish				
Not at all/once weekly	61.6 (125)	52.9 (45)	67.2 (129)	70.6 (60)
Over three times weekly/every day	38.4 (78)	47.1 (41)^*^	32.8 (63)	29.4 (26)
Eggs				
Not at all/once weekly	72.4 (147)	70.6 (60)	68.8 (132)	74.1 (63)
Over three times weekly/every day	27.6 (56)	29.4 (26)	31.3 (60)	25.9 (23)
Cured meat				
Not at all/once weekly	47.1 (97)	65.1 (56)**	49.9 (94)	48.8 (42)
Over three times weekly/every day	52.2 (106)	34.9 (30)	51.0 (98)	51.2 (44)
Vegetables				
Not at all/once weekly	18.4 (38)	8.1 (7)**	18.2 (35)	16.5 (14)
Over three times weekly/every day	80.1 (165)	91.9 (79)	81.8 (157)	83.5 (72)
Fresh fruit				
Not at all/once weekly	21.8 (45)	11.6 (10)**	19.8 (38)	11.6 (10)
Over three times weekly/every day	76.7 (158)	88.4 (76)	80.2 (154)	88.4 (76)
Sweets				
Not at all/once weekly	55.2 (112)	56.5 (48)	43.2 (83)	47.1 (40)
Over three times weekly/every day	44.8 (91)	43.5 (38)	56.8 (109)	52.9 (46)
Junk food				
Not at all/once weekly	75.5 (156)	86.9 (74)^*^	72.9 (140)	75.5 (65)
Over three times weekly/every day	24.3 (50)	13.1 (12)	27.1 (52)	24.5 (21)

* *p* < 0.05; **p < 0.01.

The proportion of participants who reported daily use of alcohol significantly decreased in the experimental group, from 29.1 to 10.5% (*p* < 0.01), whereas no statistically significant change was noted in the controls (from 29.7% at baseline to 24.1% at 1-year follow-up) (Fig. 1).

**Fig. 1:**
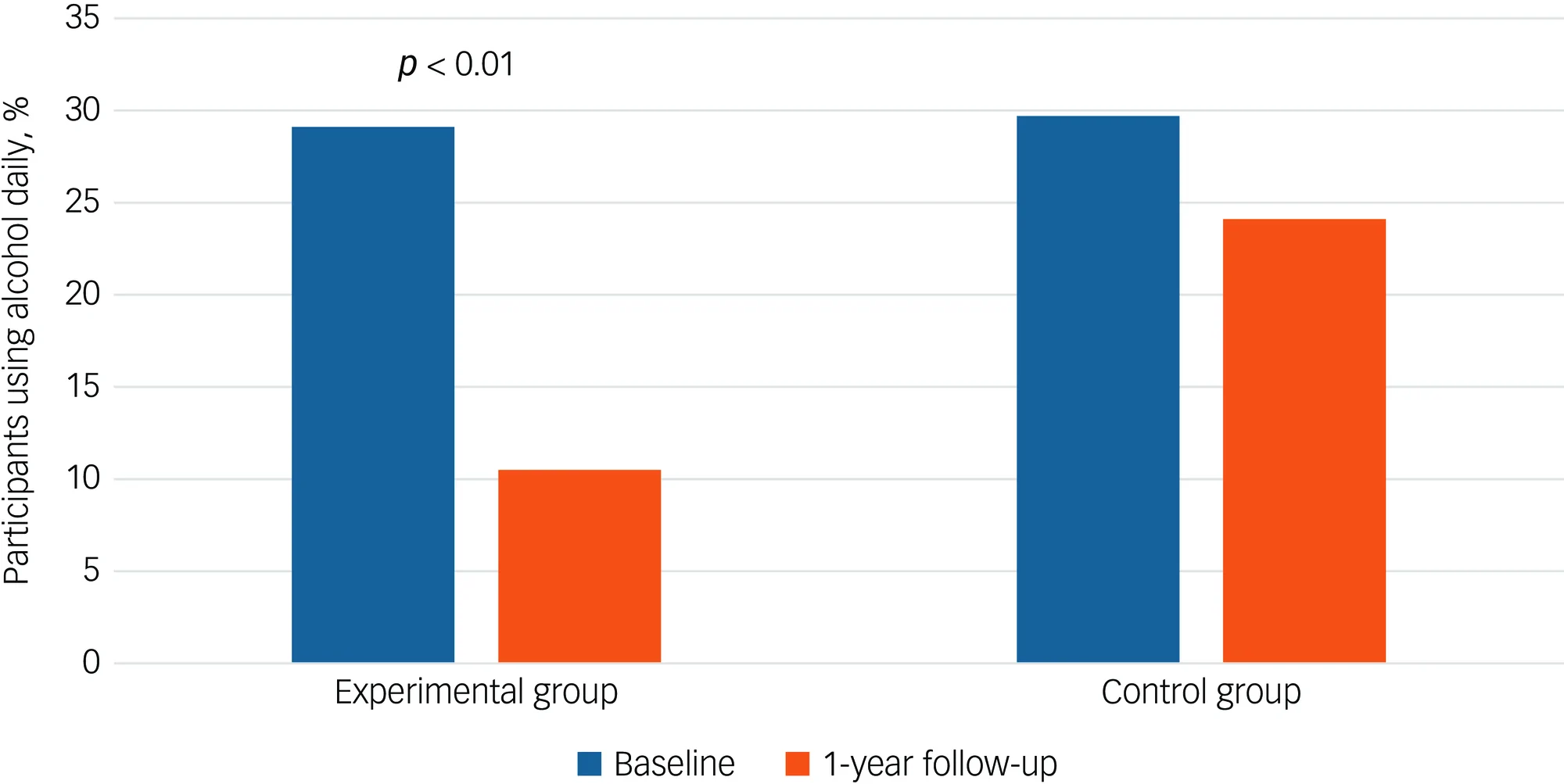
Changes in daily alcohol use between baseline and 1-year follow-up in the intervention and control groups.

The GEE analyses validated in part the beneficial impact of the trial intervention on lifestyle habits. Specifically, participants who underwent the experimental treatment were more likely to achieve higher total METs on the IPAQ after 1 year (odds ratio 1.43, 95% CI 1.08–1.89; *p* < 0.01), walking METs (odds ratio 1.50, 95% CI 1.18–1.90; *p* < 0.001) and moderate activity METs (odds ratio 1.85, 95% CI 1.24–2.77; *p* < 0.01) (Table 3). Other factors associated with increased METs were PSP total score (which was positively associated with higher walking METs at baseline (odds ratio 1.01, 95% CI 1.00–1.02; *p* < 0.05)), BPRS depression/anxiety subscale (which was inversely associated with moderate activity METs (odds ratio 0.14, 95% CI 0.01–0.68; *p* < 0.05)) and BPRS psychotic symptoms subscale (also inversly related to moderate activity (odds ratio 0.90, 95% CI 0.84–0,99; *p* < 0.05)). In the experimental group, vigorous activity METs significantly increased from baseline to follow-up (*p* < 0.01), whereas no significant change was observed in the control group. In the whole sample, participants with higher BMI (odds ratio 0.99, 95% CI 0.98–1.00; *p* < 0.05), higher BPRS ‘anergia’ scores (odds ratio, 95% CI 0.08–0.26; *p* < 0.05) or higher psychotic symptom scores (odds ratio 0.79, 95% CI 0.68–0.92; *p* < 0.01) were less likely to increase their vigorous activity METs.

**Table 3:** Generalised estimating equation (GEE) models – physical exercise, diet and daily alcohol use^a^ Table 3 long description.

	IPAQ total METS	IPAQ walking METS	IPAQ moderate METS	IPAQ vigorous METS	Red meat	Cured meat	Fish	Fresh fruit	Junk food	Vegetables	Daily use of alcohol
	OR (95% CI)	OR (95% CI)	OR (95% CI)	OR (95% CI)	OR (95% CI)	OR (95% CI)	OR (95% CI)	OR (95% CI)	OR (95% CI)	OR (95% CI)	OR (95% CI)
Experimental treatment	1.43 (1.08–1.89)**	1.50 (1.18–1.90)***	1.85 (1.24–2.77)**	1.33 (0.96–1.86)	1.15 (0.88–1.49)	1.04 (0.82–1.32)	1.67 (1.45–1.97)^*^	1.36 (1.05–1.76)^*^	0.81 (0.63–.99)^*^	1.91 (1.56–1.96)^*^	0.70 (0.52–0.95)^*^
Body mass index	0.98 (0.95–1.01)	0.98 (0.96–1.01)	0.99 (0.97–1.00)	0.99 (0.98–1.00)^*^	1.00 (0.97–1.04)	0.99 (0.96–1.04)	0.97 (0.95–1.0)	1.00 (0.97–1.03)	1.00 (0.97–1.02)	1.03 (0.98–1.07)	0.98 (0.92–1.04)
BPRS depression/anxiety subscale	1.01 (0.98–1.05)	0.97 (0.94–1.01)	0.14 (0.01–0.68)^*^	1.08 (0.90–1.26)	1.00 (0.97–1.04)	1.06 (0.99–1.14)	1.02 (0.99–1.06)	1.09 (1.02–1.15)	1.03 (0.97–1.10)	1.00 (0.93–1.08)	0.93 (0.78–1.12)
BPRS anergia	0.98 (0.94–1.03)	1.01 (0.98–1.04)	0.92 (0.80–1.05)	0.17 (0.08–0.26)**	1.00 (0.92–1.08)	1.03 (0.98–1.06)	1.07 (0.98–1.16)	0.97 (0.86–1.07)	0.99 (0.91–1.08)	0.97 (0.97–1.04)	0.91 (0.83–0.99)^*^
BPRS psychotic symptoms	0.98 (0.92–1.06)	1.04 (0.96–1.12)	0.90 (0.84–0.99)^*^	0.79 (0.68–0.92)**	1.08 (0.97–1.19)	1.00 (0.87–1.13)	0.93 (0.85–1.02)	1.11 (0.97–1.27)	0.97 (0.83–1.12)	1.06 (0.96–1.17)	1.19 (0.91–1.56)
BPRS hyperactivity symptoms	1.10 (1.01–1.20)	1.03 (0.93–1.13)	1.15 (0.94–1.40)	1.45 (1.14–1.86)	1.05 (0.93–1.19)	0.92 (0.83–1.03)	0.98 (0.87–1.10)	0.98 (0.84–1.15)	1.04 (0.88–1.23)	1.03 (0.94–1.12)	0.98 (0.73–1.32)
BPRS hostility symptoms	0.97 (0.91–1.04)	1.00 (0.94–1.08)	0.91 (0.79–1.05)	0.93 (0.83–1.04)	1.01 (0.88–1.49)	0.91 (0.83–0.99)^*^	0.89 (0.81–0.99)^*^	1.00 (0.99–1.02)	1.12 (1.03–1.21)^*^	1.01 (0.99–1.03)	0.95 (0.76–1.19)
PSP, total score	1.01 (1.01–1.019)^*^	1.01 (1.00–1.02)^*^	1.01 (1.00–1.02)	1.00 (0.99–1.01)	1.00 (0.99–1.01)	1.00 (1.00–1.01)	1.00 (0.98–1.01)	1.15 (0.90–1.45)	0.99 (0.97–1.02)	1.01 (1.00–1.03)	0.87 (0.39–1.03)
CIRS, comorbidity index	1.06 (0.95–1.17)	1.12 (1.00–1.26)^*^	0.84 (0.48–1.50)	0.41 (0.09–1.94)	0.88 (0.54–1.45)	1.06 (0.89–1.34)	1.12 (0.84–1.50)	1.06 (0.45–1.45)	1.30 (0.41–4.11)	1.77 (0.47–6.70)	4.68 (1.27–7.18)^*^
CIRS, severity index	0.64 (0.39–1.89)	0.75 (0.50–1.13)	0.54 (0.18–2.77)	0.75 (0.50–1.15)	0.85 (0.69–1.04)	1.04 (0.82–1.32)	0.88 (0.66–1.16)	1.06 (0.88–1.98)	0.75 (0.52–1.06)	1.03 (0.86–1.25)	0.89 (0.60–1.32)

METs, metabolic equivalents, minutes/week; OR, odds ratio; BPRS, Brief Psychiatric Rating Scale; PSP, Personal and Social Performance; CIRS, Cumulative Illness Rating Scale.

* *p* < 0.05; **p < 0.01; ***p < 0.001.

a. GEEs have been adjusted for age, study centre, medications, cognitive functioning, gender and diagnosis type.

Concerning additional outcome measures, the GEE models verified the favourable influence of the experimental intervention on eating patterns. Specifically, those who received the intervention showed an increase in fish consumption (odds ratio 1.67, 95% CI 1.45–1.97; *p* < 0.05), fresh fruit (odds ratio 1.36, 95% CI 1.05–1.76; *p* < 0.05) and vegetables (odds ratio 1.91, 95% CI 1.56–1.96; *p* < 0.05). Moreover, the experimental intervention was related to a reduction of junk food (odds ratio 0.81, 95% CI 0.63–0.99; *p* < 0.05) and daily alcohol use (odds ratio 0.70, 95% CI 0.52–0.95; *p* < 0.05). Additionally, we found that intake of fish (odds ratio 0.89, 95% CI 0.81–0.99; *p* < 0.05) and cured meat (odds ratio 0.91, 95% CI 0.83–0.99; *p* < 0.05) was negatively influenced by BPRS hostility symptoms, which were linked to heightened consumption of junk food (odds ratio 1.12, 95% CI 1.03–1.21; *p* < 0.05). Daily alcohol use was also influenced by the BPRS anergia subscale (odds ratio 0.91, 95% CI 0.83–0.99; *p* < 0.05), whereas CIRS comorbidity correlated with a significant increase in daily alcohol use (odds ratio 4.68, 95% CI 1.27–7.18; *p* < 0.05) (Table 3).

Overall, the intervention was associated with healthier eating patterns, with participants increasing their intake of fish, fruit and vegetables and reducing junk food consumption.

## Discussion

### Main findings

Our results support the main study hypothesis that people with SMI may benefit from a behavioural lifestyle intervention in terms of increasing physical activity, eating healthier food and decreasing use of alcohol after 1 year. The effectiveness of the LIFESTYLE intervention in promoting healthy living behaviours by reducing participants’ intake of junk food and increasing vegetable intake and physical exercise was already evident at 6 months,^20^ along with a significant improvement in BMI and waist circumference.^12^ In this paper we demonstrate that positive effects of the LIFESTYLE intervention are maintained over a longer follow-up (1 year), with an additional positive impact in reducing the daily use of alcohol.

In our study, at baseline, just a few patients participated in enough physical activity, and the majority were inactive. Moreover, their dietary habits were characterised by low intake of healthy foods, such as fruit and vegetables, and high intake of potentially harmful food, such as red meat, processed meat and junk food. Our experimental LIFESTYLE intervention had a solid positive effect in improving physical activity levels and healthy dietary habits, suggesting that comprehensive lifestyle interventions are highly effective approaches to reduce health disparities in people with SMI, to recover their physical health, reduce chronic diseases and foster quality of life.^21^ Although our study did not assess chronic disease outcomes or quality of life, previous research has shown that healthier lifestyle behaviours are associated with lower cardiometabolic risk and improved quality of life.^22^ These potential benefits, however, fall outside the scope of the present analysis.

More specifically, treated patients showed substantial improvements in various aspects of physical activity, including METs expended on vigorous and moderate activity and walking. Previous studies showed a minimal impact of multicomponent interventions on levels of physical activity. Our results are in line with previous multicomponent lifestyle interventions in people with SMI, which have shown improvements in dietary behaviours and physical activity, although effects were frequently modest or not sustained beyond the short term.^23^ Compared with these studies, our trial demonstrated significant changes sustained at 12 months, supporting the added value of a structured, multidimensional approach. The discrepancy between our findings and those in the literature may be due to some peculiarities of our intervention; first, the delivery of the intervention in small groups, which facilitated peer interaction and improved social support and shared experiences. This peer interaction may have fostered a sense of accountability and motivation among participants, helping them to adopt and maintain healthy lifestyle behaviours. Second, the inclusion of a motivational component seems to have played a crucial role in the effectiveness of the intervention. Mental health professionals were able to foster a profound commitment to lifestyle changes by actively reinforcing participants’ motivation to change. Additionally, the incorporation of problem-solving techniques enabled participants to address and overcome real-life obstacles faced when they tried to modify their behaviour. This approach not only empowered participants, but also provided practical skills to sustain changes over time. These innovative elements contributed to the better outcomes observed in our study, highlighting the need to provide tailored approaches to achieve significant improvements in physical care in individuals with SMI.^24,25^

Our findings confirm the negative association between psychiatric symptoms and levels of physical activity. In particular, high levels of anergia and positive symptoms in the psychosis subgroups were associated with decreased METs. In their systematic review, Tarpada & Morris^26^ showed that the presence of hallucinations and delusions might reduce individuals’ participation in physical activity, by confining them to their homes, discouraging them from going out for a walk, instructing them to engage in activities that interfere with exercise, or causing distraction and distress. It is also noteworthy that anergia, closely associated with dopamine depletion, correlates with reduced vigorous exercise in animal models,^27^ supporting the hypothesis that the illness itself or dopamine antagonist medications can adversely influence physical activity in people with SMI. Additionally, although it is not entirely clear how second-generation antipsychotics cause metabolic dysfunction, we know that these medications, particularly olanzapine and clozapine, promote metabolic alterations, probably by interfering with adiponectin and its blood levels, thus worsening patients’ metabolic profile.^28^

The GEE analyses confirmed that anxiety/depressive symptoms correlate with METs. Other authors^29^ have already documented the negative correlation between anxiety and depression and physical activity; these symptoms should be carefully considered in clinical practice in order to improve patients’ lifestyle and increase their life expectancy.^11^

Interestingly, in the control group we found a significant reduction in overall METs over time. This finding could be due to the fact that people with SMI tend to progressively reduce their levels of physical activity, mainly owing to persistent psychiatric symptoms and lack of intrinsic motivation. This finding further highlights the need to provide routine interventions targeting physical activity in these patients.

Another interesting result is the correlation between psychosocial functioning and walking METs. This rather new finding highlights the importance of assessing patients’ psychosocial functioning in routine care, which goes beyond mere symptom reduction and aligns with a recovery-oriented approach to mental health,^30,31^ suggesting that interventions focused on the improvement of patients’ psychosocial functioning could indirectly increase their levels physical activity, potentially facilitating an overall better quality of life.^32^

We also found a significant improvement in dietary habits in participants receiving the experimental intervention. Increased intake of healthy food, such as fish, fresh fruit and vegetables, and decreased intake of red meat, cured meat, junk food and alcohol, are maintained at 1-year follow-up.

Enhancing diet, often challenging for individuals with SMI, is crucial for boosting their physical health. Nutrition plans high in vitamins, minerals, fibre and polyphenols can enhance mental health by reducing the intake of harmful and pro-inflammatory foods, such as red meats and refined carbohydrates, which are linked to various psychiatric symptoms.^33^ From a biological perspective, dietary changes that reduce the intake of pro-inflammatory foods may attenuate systemic low-grade inflammation, which has been implicated in the pathophysiology of several psychiatric disorders. Elevated inflammatory markers, such as cytokines (e.g. interleukin 6, tumour necrosis factor alpha and C-reactive protein), have been associated with depressive and psychotic symptoms, and their reduction has been linked to improvements in mood and cognition.^34^ Therefore, promoting an anti-inflammatory dietary profile may be one plausible mechanism underlying the clinical benefits observed.

### Comparison with previous studies

Previous trials on the effect of behavioural interventions on dietary habits of people with SMI reported highly heterogeneous results. Parletta et al^35^ reported significant improvements in diet quality and mental health outcomes at 6 months, but only a few other clinical studies reported any changes in dietary behaviours.

The strong effectiveness of the LIFESTYLE intervention in maintaining positive dietary habits after 1 year, even higher than the 6-month results,^20^ may be attributed to several factors, including the structured approach, dedicated sessions on healthy diet components and on balancing daily intake of nutrients, the strong focus on the Mediterranean diet, the provision of practical examples on how to improve dietary habits, the adoption of problem-solving techniques, and the duration of the intervention compared with other trials.^13^

Last, the decrease in daily alcohol use observed in the experimental group is particularly noteworthy, given the well-documented negative effect of alcohol on physical and mental health.^10^ Our results showing that the LIFESTYLE intervention is effective in reducing daily alcohol use suggest that these approaches should be integrated in routine care of patients with comorbid mental and alcohol use disorders.

Notably, higher psychosocial functioning (PSP) was associated with greater physical activity (walking METs), underscoring the clinical relevance of integrating functional rehabilitation with lifestyle interventions.

### Strengths and limitations

Our study highlights several strengths of the LIFESTYLE intervention, making it innovative and suitable for routine care of people with SMI. These include: (a) a motivational component, crucial to sustaining long-term behavioural change; (b) a comprehensive focus on multiple lifestyle domains (diet, sedentary behaviour, physical activity, tobacco use, sexual health, sleep, circadian rhythms and medication adherence); (c) the requirement of only brief training for mental health professionals; (d) a transdiagnostic applicability across different psychiatric conditions; (e) the fact that it can be delivered by any trained mental health professional, thus reducing costs and enhancing scalability; and (f) a group format that fosters peer support and sharing of experiences beyond illness-related issues.

Among the principal strengths of our study is the comprehensive analysis of multiple outcome measures, as detailed in the published protocol.^15^ This allowed us to demonstrate multiple positive benefits, both on participants’ lifestyle behaviours and on physical health, over a follow-up of 1 year, which is a strength itself. These results highlight the crucial role of structured and multidimensional approaches in ensuring sustained outcomes in mental health. In contrast, many psychosocial interventions are evaluated with only short follow-up periods, so evidence on their long-term sustainability remains limited.^36^

However, the study itself also has several limitations, including the high drop-out rate and the use of self-reported instruments. The relatively high drop-out rate is a significant limitation that may affect the generalisability of our findings. Attrition analyses did not reveal significant differences between completers and those who dropped out in terms of baseline sociodemographic, clinical or anthropometric/metabolic characteristics.^37^ Nevertheless, the possibility of attrition bias cannot be fully excluded. Future studies should incorporate strategies to enhance retention.

The reliance on self-reported measures for dietary habits and physical activity may have introduced recall and desirability biases. These are common issues in studies on the efficacy of psychosocial interventions that we tried to overcome as much as possible. On the positive side, we used electronic reminders and dedicated staff to increase retention rate; the use of objective measures for dietary habits and physical activity reduced cost-effectiveness but increased the generalisability of the intervention to real-world settings; standardised and validated tools such as the IPAQ, along with a structured data collection, and clear, short reference periods, helped us to improve accuracy and reduce social pressure to minimise desirability and recall biases; finally, even though the follow-up was not that long, it was longer than usual in such trials. Another potential limitation is the shorter duration of the intervention delivered to the control group. Nevertheless, most RCTs on psychosocial treatments assess the effectiveness of experimental interventions against treatment as usual, a waiting list (i.e. no active comparator) or no intervention at all. Therefore, we regard the comparison between two active interventions as a strength of our protocol.

Given these limitations, future studies should consider the use of emerging technologies, such as wearable devices and digital tools (for example apps for monitoring diet, physical activity or alcohol consumption), to provide more objective and real-time assessments. We did not identify relevant external campaigns or social changes during the study period that could have influenced participants’ behaviour. However, future studies should explicitly consider and monitor such potential confounders. In our study, the inclusion of a control group helped isolate the effect of the intervention.

Additional potential limitations involve not assessing the effects of psychiatric medications on outcomes. We attempted to mitigate this by adjusting the GEE for medication use and by selecting participants who were in a stable phase of their illness. Another limitation might be the use of the BPRS for assessing levels of psychopathology, which may not be the ideal standard for determining symptom type and severity. We decided to use the BPRS because it is widely used in clinical and research contexts and thus our results can be generalisable to other contexts. A further limitation is that the intervention was grounded in Mediterranean dietary patterns, which may reduce its direct applicability in non-Mediterranean settings. Nevertheless, the core principles of increasing fruit, vegetable and fish consumption and reducing processed and high-fat foods are broadly transferable and could be adapted to different cultural contexts.

The single-country setting may limit international generalisability. However, the behavioural framework and nutritional principles are adaptable to different health systems and food cultures.

### Clinical and research implications

Our findings support the efficacy of structured lifestyle interventions for enhancing physical activity and dietary behaviours and for reducing alcohol use in people with SMI. The improvements reported in treated participants suggest that comprehensive lifestyle intervention can reduce health disparities and enhance well-being in people with SMI.^38,39^

Our findings also support the translation of similar interventions into clinical practice and show the need to provide advice on physical activity and dietary regimens to people with SMI as part of their treatment plan. Studies on the long-term sustainability of these improvements, elucidating the most active components of lifestyle interventions, should be a focus of future research in order to maximise their benefits.

## Supporting information

Luciano et al. supplementary material 1 Luciano et al. supplementary material

Luciano et al. supplementary material 2 Luciano et al. supplementary material

Luciano et al. supplementary material 3 Luciano et al. supplementary material

Luciano et al. supplementary material 4 Luciano et al. supplementary material

## Data Availability

The data supporting the findings of this study are available from the corresponding author on reasonable request.

## Supplementary material

The supplementary material is available online at https://doi.org/10.1192/bji.2025.10076

## Table 1 Long description

The table compares physical activity levels between experimental and control groups over a one-year period. It has six rows and five columns. The columns are labeled as Experimental group Baseline (T0) METs, minutes/week (n = 206), 1-year follow-up (T2) METs, minutes/week (n = 87), Control group Baseline METs, minutes/week (n = 195), and 1-year follow-up (T2) METs, minutes/week (n = 83). The rows are labeled as Vigorous activity, Moderate activity, Walking, and Total physical activity. Each row contains mean (s.d.) and median (IQR) values for the respective activity categories and groups. The table shows the changes in physical activity levels over the one-year period for both groups.

Navigate back to Table 1.

## Table 2 Long description

A table comparing eating patterns between experimental treatment and control group at baseline and 1-year follow-up. The table has 12 rows and 8 columns. Column headers are: Experimental treatment Baseline (T0) (n = 206), 1-year follow-up (T2) (n = 87), Control group Baseline (T0) (n = 195), 1-year follow-up (T2) (n = 86). Row labels are: Bread, Pasta and rice, Red meat, White meat, Legumes, Fish, Eggs, Cured meat, Vegetables, Fresh fruit, Sweets, Junk food. Each row contains percentages and counts for two categories: Not at all/once weekly and Over three times weekly/every day. Notable trends include increased consumption of red meat, fish, fresh fruit, and vegetables in the experimental group, and no significant changes in the control group.

Navigate back to Table 2.

## Table 3 Long description

A table with 12 rows and 12 columns comparing the impact of various factors on physical activity and diet. The columns are IPAQ total METS, IPAQ walking METS, IPAQ moderate METS, IPAQ vigorous METS, Red meat, Cured meat, Fish, Fresh fruit, Junk food, Vegetables, and Daily use of alcohol. The rows are Experimental treatment, Body mass index, BPRS depression/anxiety subscale, BPRS anergia, BPRS psychotic symptoms, BPRS hyperactivity symptoms, BPRS hostility symptoms, BPRS total score, CIRS total score, CIRS comorbidity index, and CIRS severity index. Each cell contains an odds ratio and a 95% confidence interval. Notable trends include higher odds ratios for IPAQ total METS, IPAQ walking METS, and IPAQ moderate METS in the experimental treatment group, and lower odds ratios for IPAQ moderate METS and IPAQ vigorous METS in participants with higher BMI and higher BPRS scores.

Navigate back to Table 3.
