# Supplementary material for: One-year efficacy of the LIFESTYLE intervention in improving diet, physical activity and reducing alcohol use in overweight people with severe mental illness: multicentre randomised controlled trial
Source: BJPsych Int. 2025 Dec 1;23(4):200–11. doi: 10.1192/bji.2025.10076 (PMC13732257; doi:10.1192/bji.2025.10076)
Supplement: Supplementary file 1 [file S2056474025100767sup002.docx]

**Figure 2.** Changes in Physical Activity METS - minutes/week between baseline and one-year follow-up in the two groups
