# Supplementary material for: One-year efficacy of the LIFESTYLE intervention in improving diet, physical activity and reducing alcohol use in overweight people with severe mental illness: multicentre randomised controlled trial
Source: BJPsych Int. 2025 Dec 1;23(4):200–11. doi: 10.1192/bji.2025.10076 (PMC13732257; doi:10.1192/bji.2025.10076)
Supplement: Supplementary file 2 [file S2056474025100767sup003.docx]

The following is a list of authors who have contributed to the development of the work titled "One-year efficacy of the LIFESTYLE intervention on improving diet, physical activity and reducing alcohol use in patients with severe mental disorders: results from a multicentric randomized controlled trial." Therefore, they should be recognized with authorship credit, as “LIFESTYLE Working Group”.

**LIFESTYLE Working Group:**

Salvatore Cipolla, Claudia Toni, Vincenzo Giallonardo, Valeria Del Vecchio (Department of Psychiatry, University of Campania “L. Vanvitelli”, Naples); Piergiuseppe Di Palo, Pierluigi Selvaggi (Department of Basic Medical Science, Neuroscience and Sense Organs, University of Bari “Aldo Moro”); Giacomo Marenco, Gianluca Serafini (Section of Psychiatry, Department of Neuroscience, Ophthalmology, Genetics and Infant-Maternal Science, University of Genoa); Rodolfo Rossi, Francesca Pacitti (Section of Psychiatry, Department of Biotechnological and Applied Clinical Sciences, University of L'Aquila); Benedetta Nardi, Chiara Bonelli (Department of Clinical and Experimental Medicine, University of Pisa); Cinzia Niolu, Michele Ribolsi (Department of System Medicine, University of Rome Tor Vergata).
