# Supplementary material for: One-year efficacy of the LIFESTYLE intervention in improving diet, physical activity and reducing alcohol use in overweight people with severe mental illness: multicentre randomised controlled trial
Source: BJPsych Int. 2025 Dec 1;23(4):200–11. doi: 10.1192/bji.2025.10076 (PMC13732257; doi:10.1192/bji.2025.10076)
Supplement: Supplementary file 3 [file S2056474025100767sup004.docx]

**Table S1.** Socio-demographic and clinical characteristics of the sample

|  | Experimental group  (N=206) | Control group  (N=195) |
| --- | --- | --- |
| Gender, female, % (N) | 55.3 (114) | 59 (115) |
| Age, M (sd) | 45.9 (11.6) | 45.3 (12.1) |
| Living situation, with partner yes % (N) | 47.1 (97) | 48.2 (94) |
| Years of education, M (sd) | 11.7 (2.6) | 11.7 (3.1) |
| Employed, yes, % (N) | 37.6 (77) | 33.8 (66) |
| Diagnosis, % (N)  Bipolar disorder  Major depression  Psychotic disorder | 43.2 (89)  24.8 (51)  32.0 (66) | 43.6 (85)  29.2 (57)  27.2 (53) |
| Years in charge to the mental health service, M (sd) | 68.7 (81.5) | 74.5 (84.0) |
| Duration of illness, M (sd) | 16.2 (11.7) | 16.4 (22.4) |
| Suicide attempts, M (sd) | 1.5 (1.2) | 2.0 (1.6) |
| BPRS, Anergia, M (sd) | 7.7 (3.1) | 7.6 (3.1) |
| BPRS, Psychotic symptoms, M (sd) | 5.3 (2.0) | 5.5 (2.1) |
| BPRS, Depressive/anxiety symptoms, M (sd) | 8.7 (3.0) | 8.9 (3.2) |
| BPRS, Hyperactivity sympoms, M (sd) | 4.7 (1.9) | 4.8 (1.8) |
| B-MCCB, BACS Symbol coding, M (sd) | 34.5 (14.2) | 34.5 (13.5) |
| B-MCCB, Animal naming, M (sd) | 18.2 (5.7) | 17.5 (5.1) |
| B-MCCB Trial making test A, M (sd) | 52.8 (30.5) | 51.9 (26.6) |
| IPAQ, Low PA, yes % (N) | 54.4 (111) | 59.1 (114) |
| IPAQ, Moderate PA, yes % (N) | 29.4 (60) | 24.9 (48) |
| IPAQ, High PA, yes % (N) | 16.2 (33) | 16.1 (31) |
| PSP, Total score, M (sd) | 66.5 (14.8) | 64.8 (15.5) |

MANSA: Manchester Short Assessment of Quality of Life; ISMI: Internalized Stigma of Mental Illness scale, MMAS: Morisky Medication Adherence Scale; B-MCCB: Brief MATRICS Consensus Cognitive Battery; PSP: Personal and Social Performance Scale; BPRS: Brief Psychiatric Rating Scale; BACS: Brief Assessment of Cognition in Schizophrenia; IPAQ: International Physical Activity Questionnaire; PA: Physical Activity
