# Supplementary material for: One-year efficacy of the LIFESTYLE intervention in improving diet, physical activity and reducing alcohol use in overweight people with severe mental illness: multicentre randomised controlled trial
Source: BJPsych Int. 2025 Dec 1;23(4):200–11. doi: 10.1192/bji.2025.10076 (PMC13732257; doi:10.1192/bji.2025.10076)
Supplement: Supplementary file 4 [file S2056474025100767sup001.doc]

**CONSORT 2010 Flow Diagram**

**Allocation**

**Analysis**

**Follow-Up**

**Enrollment**

Assessed for eligibility (n= 530) )

Excluded (n= 129)

♦  Not meeting inclusion criteria (n= 98)

♦  Declined to participate (n= 41)

♦  Other reasons (n= 0)

Analysed (n= 87)

Lost to follow-up (changes in the local mental health center; severe worsening of mental health status; low motivation to complete the follow-up assessments) (n= 7)

Allocated to intervention (n= 206)

♦ Received allocated intervention (n= 94)

Lost to follow-up (n= 0)

Allocated to control group (n= 195)

♦ Received allocated intervention (n= 83)

Analysed (n= 83)

Randomized (n= 402 )
